# Psychiatric help for adolescents with autoaggression in Ukraine population

**DOI:** 10.1192/j.eurpsy.2022.1118

**Published:** 2022-09-01

**Authors:** K. Zhyvaho, A. Bursa

**Affiliations:** Bohomolets National Medical University, Psychiatry And Narcology, Kyiv, Ukraine

**Keywords:** Suicide, criteria, self-harm, prevention

## Abstract

**Introduction:**

During several years in Ukraine have been actualized problem with autoaggressive behavior amoung young people. Due to not enough support at ambulatory psychiatric and social systems these patients have hospitalizations. And its duration could be for month and longer. The problem seems like if hospitalization can be ling, will be it affective while there are no community support after it. Even having good results can not give long “remission” because patients come to the same family/social situation.

**Objectives:**

Examine autoaggressive behavior in adolescents and find criteria for hospitalization for this category.

**Methods:**

We took 173 patients with autoaggressive behavior at age 18-25. We formed theory for research that, on our opinion, include information that give chance to find criteria while hospitalization isn’t recommended.

**Results:**

First results have shown a high level of comorbidity personality disorders with neurotic and depressive disorders. High levels of self-harm are associated with episodes of sexual and psychological abuse and characterized with trauma. The next parts of the research will show deep indications for in- and out-patient treatment.

**Conclusions:**

Criteria for hospitalization adolescents with self-harm are hard to form because of differences of reasons, comorbidities and risk of suicide among adolescents. But not all in-patient treatment gives expected results. Mostly it can work like a traumatic experience on this group. Scientific research can help to make the psychiatric systems friendly to adolescents with complex problems. The authors have not supplied a conflict-of-interest statement

**Disclosure:**

No significant relationships.

